# A Novel *PHEX* Mutation in Japanese Patients with X-Linked Hypophosphatemic Rickets

**DOI:** 10.1155/2015/301264

**Published:** 2015-03-15

**Authors:** Tetsuya Kawahara, Hiromi Watanabe, Risa Omae, Toshiyuki Yamamoto, Tetsuya Inazu

**Affiliations:** ^1^Division of Endocrinology and Metabolism, Department of Internal Medicine, Niigata Rosai Hospital, Niigata 9428502, Japan; ^2^Department of Clinical Laboratory, Niigata National Hospital, Niigata 9458585, Japan; ^3^Department of Pharmacy, College of Pharmaceutical Sciences, Ritsumeikan University, Shiga 5258577, Japan; ^4^Tokyo Women's Medical University, Institute of Integrated Medical Sciences, Tokyo 1620054, Japan; ^5^Department of Clinical Research, Saigata National Hospital, Niigata 9493193, Japan

## Abstract

X-linked hypophosphatemic rickets (XLH) is a dominant inherited disorder characterized by renal phosphate wasting, aberrant vitamin D metabolism, and abnormal bone mineralization. Inactivating mutations in the gene encoding phosphate-regulating gene with homologies to endopeptidases on the X chromosome (*PHEX*) have been found to be associated with XLH. Here, we report a 16-year-old female patient affected by hypophosphatemic rickets. We evaluated her serum fibroblast growth factor 23 (FGF23) levels and conducted sequence analysis of the disease-associated genes of FGF23-related hypophosphatemic rickets: *PHEX*, *FGF23*, dentin matrix protein 1, and ectonucleotide pyrophosphatase/phosphodiesterase 1. She was diagnosed with XLH based on her clinical features and family history. Additionally, we observed elevated FGF23 levels and a novel *PHEX* exon 9 mutation (c.947G>T; p.Gly316Val) inherited from her father. Although bioinformatics showed that the mutation was neutral, Gly316 is perfectly conserved among humans, mice, and rats, and there were no mutations in other FGF23-related rickets genes, suggesting that *in silico* analysis is limited in determining mutation pathogenicity. In summary, we present a female patient and her father with XLH harboring a novel *PHEX* mutation that appears to be causative of disease. Measurement of FGF23 for hypophosphatemic patients is therefore useful for the diagnosis of FGF23-dependent hypophosphatemia.

## 1. Introduction

X-linked hypophosphatemic rickets (XLH; OMIM number 307800) is the most common genetic disorder of renal phosphate wasting, with an approximate prevalence of 1 in 20,000 [1]. The clinical features of this X-linked dominant disease include short stature, bone pain, enthesopathy, and lower extremity deformities from rickets and osteomalacia. The disease is only partially corrected by treatment with high doses of phosphate and 1,25-dihydroxyvitamin D_3_ (25-(OH)_2_D_3_) [2, 3].

XLH results from mutations in the phosphate-regulating gene with homologies to endopeptidases on the X chromosome (*PHEX*) [4]. Plasma concentrations of the phosphaturic hormone fibroblast growth factor 23 (FGF23) are reported to be elevated in most affected individuals [5, 6]. Furthermore, FGF23 is overexpressed in the bone of the Hyp mouse, an animal model of XLH, suggesting that increased FGF23 expression is the likely cause of the clinical XLH phenotype [7]. Hypophosphatemic rickets and elevated serum FGF23 levels including XLH [6, 8], autosomal dominant hypophosphatemic rickets (ADHR) [6, 9], and autosomal recessive hypophosphatemic rickets 1 and 2 (ARHR1 [10, 11] and ARHR2 [12, 13]) are caused by mutations in* PHEX*,* FGF23*, dentin matrix protein 1 (*DMP1*), and ectonucleotide pyrophosphatase/phosphodiesterase 1 (*ENPP1*) genes, respectively.

The aim of this study was to investigate the etiology of patients with hypophosphatemic rickets who exhibited serum FGF23 elevation and harbored a novel* PHEX* mutation.

## 2. Case Presentation

**Figure 1:**
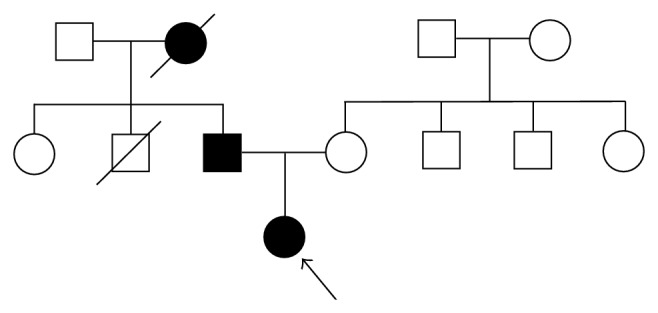
Family pedigree showing that the proband and her father have hypophosphatemic rickets and that the grandmother might have had the same disease. The death of the father's brother was unrelated to the disease. The remaining family members are healthy.

The proband was a 16-year-old Japanese girl, born at full term with a normal delivery. Her father showed short stature (−2 SD smaller than the average height for male individuals of the same age) and had a history of treatment for short stature in childhood. Her grandmother (paternal side) also exhibited short stature; however, no detailed information was available because she died 10 years previously (Figure 1). At 3 years of age, the patient was evaluated for height retardation and slight mental retardation. She was diagnosed with hypophosphatemic rickets at 4 years of age based on her clinical features, such as short stature, dental abscess, osteopenia, genu valgum, and low serum phosphate levels. At this age, her height was 90 cm (−2.24 SD) and her weight was 17 kg (+0.9 SD). Treatment with 0.5–1.5 g/day of phosphate and 0.05–0.2 *μ*g/kg/day of 1,25-(OH)_2_D_3_ was initiated to compensate for her lack of serum phosphate and vitamin D.

We measured the levels of serum minerals, FGF23, intact-parathyroid hormone (PTH), and kidney function of the patient and her parents using blood and urine samples. Ultrasound screening of the kidney was also conducted and X-rays were taken of the lower limbs. Serum FGF23 measurement was performed using the FGF-23 ELISA kit, which is a two-site enzyme-linked immunosorbent assay to measure full-length FGF23 (KAINOS Laboratories Inc., Tokyo, Japan), as described previously [6]. The institutional review board and the ethics committee of each organization approved the study. Informed written consent was obtained from all participants and volunteers.

Table 1 shows the mean laboratory data of the patient undergoing medical treatment, which included phosphate (P) 2.0 mg/dL (normal range, 3.0–4.5 mg/dL), calcium (Ca) 9.2 mg/dL (normal range, 8.7–10.2 mg/dL), alkaline phosphatase (ALP) 2374 IU/L (normal range, 100–325 IU/L), intact PTH 68.5 pg/mL (normal range, 12–72 pg/mL), 25-hydroxyvitamin D_3_ (25-(OH)D_3_) 11.0 ng/mL (normal range, 9.7–41.7 ng/mL), 1,25-(OH)_2_D_3_ 37.5 pg/mL (normal range, 20–60 pg/mL), and FGF23 400 pg/mL (normal range, 13.7–40.5 pg/mL). Urine P was 3.8 g/day (normal range, 0.4–1.2 g/day), the tubular maximum phosphate reabsorption per glomerular filtration rate was 2.1 mg/dL (normal range, 2.5–4.5 mg/dL), and the urine Ca/creatinine ratio was 0.09 (normal range, 0.05–0.25), which met the diagnostic criteria of XLH. FGF23 levels of the patient's father and mother were 68 and 29 pg/mL, respectively. Ultrasound showed normal kidney findings, while lower limb X-rays revealed a widening of the proximal tibial metaphysis with medial bowing.

To confirm the diagnosis, we conducted molecular studies, which included the direct sequencing analysis of PCR products. Genomic DNA was obtained and extracted from whole blood samples using the blood and cell genomic DNA extraction kit (Qiagen, Venlo, Netherlands). PCR amplified all 22 exons and exon-intron boundaries of* PHEX* and also all exons and exon-intron boundaries of* FGF23*,* DMP1*, and* ENPP1* to exclude ADHR, ARHR1, and ARHR2, respectively, using previously described primer pairs [1, 11, 14, 15]. Additionally, for* PHEX*, we analyzed the approximately 2 kb promoter region upstream the start codon.

**Figure 2:**
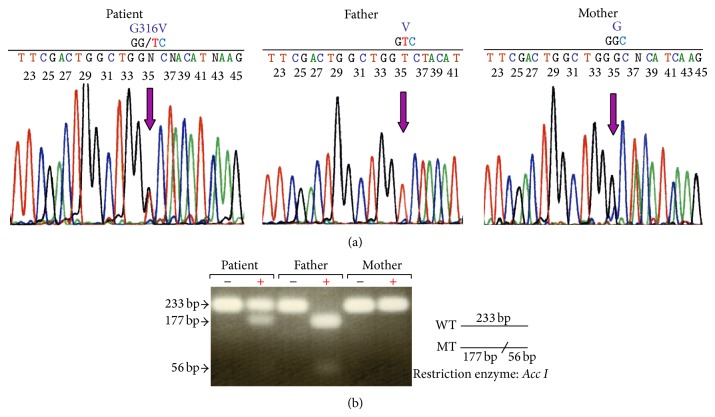
Mutation analyses. (a)* PHEX* mutation analysis in the patient's family. A missense mutation in exon 9 (c.947G>T; p.Gly316Val) of the patient was heterozygous. Her father, who exhibited short stature, carried the same mutation. Her mother has no mutation. (b) Restriction enzyme analysis. PCR products of* PHEX* exon 9 were digested with* Acc I* and separated on a 4% agarose gel. The wild-type PCR product (233 bp) lacks the restriction site, but the c.947G>T mutation introduces an* Acc I* site enabling the digestion of the product into 177 and 56 bp fragments. This analysis confirmed that the patient was heterozygous for the mutant and normal alleles and that her father also carried the mutant allele. The frequency of the mutation in 200 unrelated Japanese volunteers (100 were male and 100 were female; a total of 300 X chromosomes) was shown to be 0.33% (1/300).

We identified a mutation in exon 9 (c.947G>T; p.Gly316Val) of* PHEX* in the patient (Figure 2(a)). Additionally, we sequenced* PHEX* from her parents and showed that the mutation was inherited from her father, who also exhibited short stature (Figure 2(a)). To determine the frequency of this mutation, we carried out restriction fragment length polymorphism analysis of genomic DNA from unrelated Japanese volunteers (100 were male and 100 were female; a total of 300 X chromosomes). DNA was amplified by PCR using primers on either side of the mutation in exon 9. Amplified products were digested using* Acc I* and separated on a 4% agarose gel. Digestion of the 233 bp fragment with* Acc I* would generate fragments of 177 plus 56 bp in the presence of the mutation (Figure 2(b)). This analysis showed that only one chromosome harbored the mutation (0.33%). We further analyzed the exons and exon-intron boundaries of* FGF23*,* DMP-1*, and* ENPP1* and found no additional mutations.

When the* PHEX* mutation (Gly316Val) was identified, we conducted SIFT (http://sift.jcvi.org/) [16], PolyPhen-2 (http://genetics.bwh.harvard.edu/pph/) [17], and PROVEAN (http://provean.jcvi.org/index.php) [18] in online* in silico* analyses of Gly316 and Tyr317, which is an amino acid adjacent to Gly316. It was previously reported that the Tyr317Phe mutant protein exhibits 50–60% of* PHEX* activity [19]. SHIFT, PolyPhen-2, and PROVEN analyses predicted both variants (Gly316Val and Tyr317Phe) to be tolerated, benign, and neutral, respectively (data not shown). However, residues Gly316 and Tyr317 were shown to be perfectly conserved among humans, mice, and rats.

## 3. Discussion

The present study identified a novel heterozygous mutation in exon 9 (c. 947G>T; p.Gly316Val) of* PHEX*, which was inherited from the patient's father who exhibited short stature, so it appears to be etiological. The biochemical parameters of the female patient were more severe than those of her father, even though she had received treatment involving supplementary phosphate and 1,25-(OH)_2_D_3_. This could be explained by the required amount of phosphate decreasing with the reduction of the growth plate in her father, causing the symptoms of rickets to improve by themselves, as previously shown in adults [20]. Alternatively, some patients who responded well to treatment were able to stop receiving medication after initial therapy [21]. Therefore, the patient's father may not show such severe symptoms of rickets as the patient herself.

We identified the frequency of the mutation as 1/300 (less than 1%) in the normal Japanese population, so it was not considered to be a single nucleotide polymorphism. Although the p.Gly316Val mutation did not show pathogenicity in* in silico* analysis, Gly316 is perfectly conserved among humans, mice, and rats, so it appears to be an indispensable amino acid. Similarly, the adjacent missense mutation of p.Tyr317Phe did not show pathogenicity in* in silico* analysis, and Try317 is also perfectly conserved among these same species. In addition, the Tyr317Phe mutant protein exhibits 50–60% of the endopeptidase activity of wild-type PHEX* in vitro*, indicating that this missense mutation interferes with catalytic function [19]. Therefore,* in silico* analysis is limited in its ability to determine whether a mutation shows pathogenicity. However, because we could not investigate whether the p.Gly316Val mutant protein interferes with catalytic function and influences its activity, it remains a possibility that the mutation does not show pathogenicity. Therefore, we performed mutational screening of the* PHEX* promoter region and other genes responsible for FGF23-related rickets; this analysis identified no mutations, so we concluded that the p.Gly316Val mutation is likely to be causative of XLH.

In this study, we used the KAINOS intact assay to measure serum FGF23 levels. This is the most sensitive of all FGF23 measurement assays, which also include the Immunotopics C-terminal assay and Immunotopics intact assay [22]. The absence of a lower limit for the reference range in the C-terminal assay (≤150 RU/mL) means that we cannot distinguish between this range and lower levels. However, the KAINOS intact assay has a reference range (10–50 pg/mL), and Endo et al. proposed that its measurement of serum FGF23 levels >30 pg/mL should typically be used as a diagnostic criterion for the presence of disease caused by excess FGF23 action, such as FGF23-dependent hypophosphatemia, irrespective of medical treatment [23]. The FGF23 levels of our patient and her father were 400 and 68 pg/mL, respectively, so the data also matched the criteria, which added weight to their usefulness. Further studies examining the function of the p.Gly316Val mutation are required to extend our findings.

## Figures and Tables

**Table 1:** Laboratory data of the patient, her father, and her mother.

		Patient	Father	Mother
P	(3.0–4.5 mg/dL)	***2.0***	***2.9***	3.8
Ca	(8.7–10.2 mg/dL)	9.2	9.4	9.5
ALP	(100–325 IU/L)	***2347***	***1085***	320
Intact PTH	(12–72 pg/mL)	68.5	60.8	55.1
25-(OH)D_3_	(9.7–41.7 ng/mL)	11.0	14.5	22.6
1,25-(OH)_2_D_3_	(20–60 pg/mL)	37.5	24.1	54.0
FGF23	(10–50 pg/mL)	***400***	***68***	29
TmP/GFR	(2.5–4.5 mg/dL)	***2.1***	2.8	4.4
Urine Ca/Cre ratio	(0.05–0.25)	0.09	0.08	0.11

Values within parentheses are the normal ranges of the variant.

P, phosphate; Ca, calcium; ALP, alkaline phosphatase; intact PTH, intact parathyroid hormone; 25-(OH)D_3_, 25-hydroxyvitamin D_3_; 1,25-dihydroxyvitamin D_3_, 1,25-(OH)_2_D_3_; FGF23, fibroblast growth factor 23; TmP/GFR, tubular maximum phosphate reabsorption per glomerular filtration rate; urine Ca/Cre ratio, urine calcium/creatinine ratio.
